# Hedgehogs on the move: Testing the effects of land use change on home range size and movement patterns of free-ranging Ethiopian hedgehogs

**DOI:** 10.1371/journal.pone.0180826

**Published:** 2017-07-26

**Authors:** Mohammad A. Abu Baker, Nigel Reeve, April A. T. Conkey, David W. Macdonald, Nobuyuki Yamaguchi

**Affiliations:** 1 Department of Biological and Environmental Sciences, Qatar University, Doha, Qatar; 2 Independent consultant, 2 Paxton Gardens, Woking, United Kingdom; 3 Caesar Kleberg Wildlife Research Institute and Department of Animal, Rangeland, & Wildlife Sciences, Texas A&M University –Kingsville, Kingsville, Texas, United States of America; 4 Wildlife Conservation Research Unit, Department of Zoology, The Recanati–Kaplan Centre, University of Oxford, Tubney House, Tubney, Oxford, United Kingdom; Università degli Studi di Napoli Federico II, ITALY

## Abstract

Degradation and alteration of natural environments because of agriculture and other land uses have major consequences on vertebrate populations, particularly on spatial organization and movement patterns. We used GPS tracking to study the effect of land use and sex on the home range size and movement of a typical model species, the Ethiopian hedgehogs. We used free-ranging hedgehogs from two areas with different land use practices: 24 from an area dominated by irrigated farms (12 ♂♂, 12 ♀♀) and 22 from a natural desert environment within a biosphere reserve (12 ♂♂, 10 ♀♀). Animals were significantly heavier in the resource-rich irrigated farms area (417.71 ±12.77SE g) in comparison to the natural desert area (376.37±12.71SE g). Both habitat and sex significantly influenced the home range size of hedgehogs. Home ranges were larger in the reserve than in the farms area. Total home ranges averaged 103 ha (±17 SE) for males and 42 ha (±11SE) for females in the farms area, but were much larger in the reserve averaging 230 ha (±33 SE) for males and 150 ha (±29 SE) for females. The home ranges of individuals of both sexes overlapped. Although females were heavier than males, body weight had no effect on home range size. The results suggest that resources provided in the farms (e.g. food, water, and shelters) influenced animal density and space use. Females aggregated around high-resource areas (either farms or rawdhats), whereas males roamed over greater distances, likely in search of mating opportunities to maximize reproductive success. Most individual home ranges overlapped with many other individuals of either sex, suggesting a non-territorial, promiscuous mating. Patterns of space use and habitat utilization are key factors in shaping aspects of reproductive biology and mating system. To minimize the impacts of agriculture on local wildlife, we recommend that biodiversity-friendly agro-environmental schemes be introduced in the Middle East where the transformation from dry lands to ‘islands of fertility’ is often extreme.

## Introduction

Anthropogenic land use practices and their influence on an organism’s ecology is not only of academic interest (e.g. influencing the outcome of interspecific competition) but also key for wildlife conservation as land use change is often considered the greatest threat to terrestrial biodiversity [1, 2, 3]. In this context, it is crucial for ecologists and conservationists to understand behavioral and ecological responses of organisms to different types of land use. However, few empirical studies have investigated the responses of terrestrial mammals to changes in land use through direct comparisons of behavioral parameters between areas with different types of land use [4, 5]. This lack of knowledge is most marked in arid environments, which occupy approximately one third of the world’s terrestrial area, yet, have been neglected in terms of ecological and conservation studies of land use and its influence on local wildlife [6].

Throughout the Arabian Desert and Gulf Region, the ‘economic miracle’ of oil and natural gas extraction during the last few decades has triggered an extreme transformation of much of the formerly barren desert into urban areas, small and large-scale agricultural farms, and industrial developments [7, 8]. In Qatar, where no permanent, surface fresh water source exist, small-scale farming and livestock grazing was centered around low-land areas locally known as ‘rawdhat’ within which substantial quantities of water gather after rain events. During the last few decades, many farms have been established around those rawdhats, where natural, passive and seasonal irrigation has been replaced by modern artificial irrigation systems that provides water all year round. This dramatic change in land use is expected to influence local resource availability and distribution, which in turn is likely to influence the space use and habitat selection of various wildlife inhabiting in the region [9]. One hypothesis is that such newly-created, productive landscape in the arid environment is expected to provide water and other resources for local wildlife to flourish [10]. However, few studies have identified the effects of agricultural and other anthropogenic practices on the ecology and behavior of vertebrates in the Arabian deserts [11, 12]. Qatar has recently experienced dramatic social and physical changes through the rise in standards of living, a construction boom, and an increase in population size. These changes were accompanied by significant industrialization and development both locally, such as private farms and desert camps, and at a national level such as highways and ports [8, 13]. The country provides an excellent natural laboratory for investigating the possible effects of changes in land use on local wildlife in an arid environment.

The Ethiopian hedgehog, *Paraechinus aethiopicus* is a small nocturnal insectivore that inhabits the arid regions of North Africa, the Arabian Peninsula (including Qatar), and Southwest Asia [14]. Yet, little is known about its ecology and behavior [15, 16]. As a common solitary mammal with apparently no territoriality, *P*. *aethiopicus* is a good model species to study the influence of change in land use on mammalian space use in arid environments [16, 17].

A home range defines the area traversed by an animal in its normal activities of foraging, seeking shelter, mating and caring for young [18]. Determining this area and mapping its size and shape, is one of the essential steps in understanding patterns of resource use with a behavioral ecology perspective. Factors that influence home range size, such as land use practices, resource distribution, habitat characteristics, and population density, need more investigation [19, 20, 21]. Home range size and movement patterns are also critical for understanding the factors behind the different reproductive strategies, mating systems, and social structures that animals employ. Home range must be sufficient to provide not only an adequate food supply, but also other requirements such as shelter and potential mates [22, 23, 24].

In this paper, we report on the differences in space use patterns by free-ranging Ethiopian hedgehogs within two study sites with contrasting land use practices: a nature reserve and an area dominated by agricultural farms. Specifically, we tested for sex and habitat-specific differences in home range size and movement patterns. We used GPS telemetry to compare: **1.** home range sizes, **2.** length of daily distance travelled, and **3.** percent home range overlap for male and female hedgehogs. We used the results to deduce the changes in behavior and ecology of hedgehogs associated with recent changes in land use in arid environments.

## Materials and methods

### Study sites

**Fig 1:**
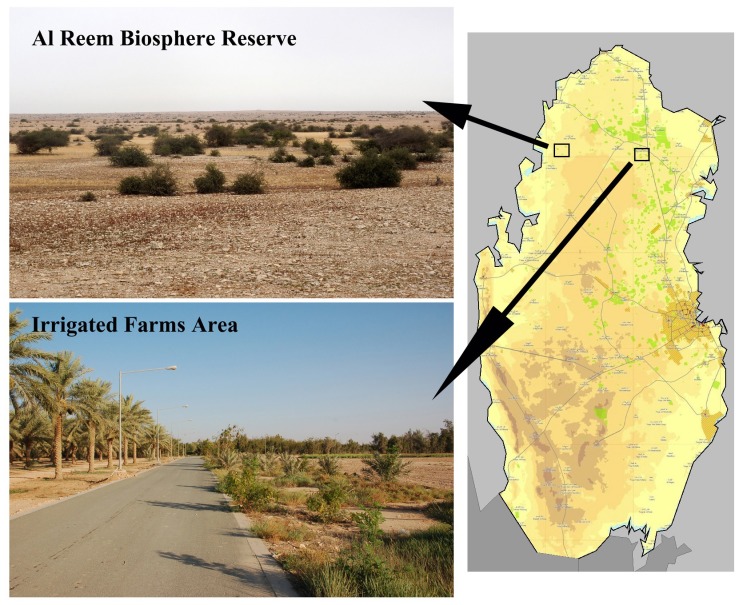
The study sites in the north of Qatar: Al Reem Biosphere Reserve and one of the irrigated farms in Rawdat Al Faras area.

The study was conducted at two sites in northern Qatar (30 km apart) with different land use practices: around Qatar University Farm (25° 48.4' N, 51° 20.8' E) and Rawdat Al Faras Agricultural Research Station (25° 49.35' N, 51° 19.95' E) (hereafter farms area), and Al Reem Biosphere Reserve (25° 53.8' N, 51° 03.00' E) (hereafter reserve area) from April to June of 2014 and 2015. The farms area consisted of c. 15 km^2^ of arid area including 11 active farms that receive regular irrigation. Each farm is fenced and partitioned into agricultural fields with asphalt roads. Plantations of date palm, olives, citrus, and other ornamental and windbreak evergreen trees (e.g. eucalyptus and pine) are found in the farms (Fig 1). Arid plains surround the farms with the surface predominantly covered by desert pavement with exposed loose gravel. The vegetation included isolated short acacia trees with some ephemeral grass patches emerging after the rains in cooler months (usually between November and March). The study sites in the reserve covered an area of c. 15 km^2^ in the western part of the Al Reem Biosphere Reserve (c. 1,190 km^2^) in the northwestern corner of Qatar. It is characterised by gravel plain ecosystems interspersed with seasonal riverbeds (run-off wadis) and low-land areas locally known as ‘rawdhat’ or ‘marab’, representing the typical pre-irrigation land use found in Qatar. The area is degraded by overgrazing by camel and sheep and accommodates a suite of small-scale agricultural settlements and recreational winter camps [13]. The majority of the reserve area is barren stone desert and rarely covered by vegetation, whilst the water run-off-systems (wadis) and rawdhats provide vegetated bush to shrubby microsystems mainly of *Astralagus spinosus*, *Lycium shawii*, and *Vachellia* (*Acacia*) *tortilis* that reach up to 2 m in height (Fig 1).

### Data collection

**Fig 2:**
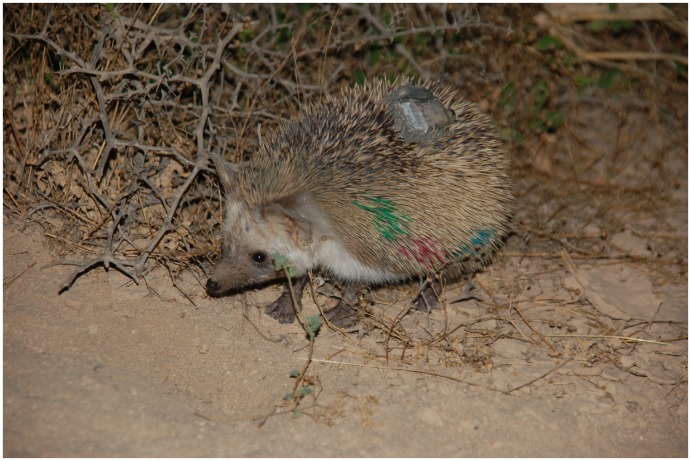
Attachment of a GPS tag to the back of a marked hedgehog.

Hedgehogs were hand-captured during the night on linear transects along led roads within the farms or dirt roads within the reserve, or accidentally captured while tracking tagged animals. All animals were individually color-marked, sexed, and weighed. Population densities were estimated using the total number of individual hedgehogs that were captured and marked during the study (minimum number alive) within the defined search areas in each site. GPS tags (8 GPS BUG and 12 GPS PinPoints, Biotrack Ltd., Wareham, UK) were glued to the spines on the backs of 46 adult hedgehogs, 24 in the farms area (12 ♂♂, 12 ♀♀) and 22 in the reserve area (12 ♂♂, 10 ♀♀) following the methods described in Warwick et al. (2006) [25] and Abu Baker et al. (2016) [17] as shown in Fig 2. A small VHF Pip radio tag (Biotrack Ltd., Wareham, UK) was attached to the GPS tag upon deployment to track the animals and retrieve the GPS tags for data download. The combined weight of the tags averaged 10-12g (c. 2.5–3.5% of the animals’ weights). Animals were tagged in groups of 4–8 at a time within both sites simultaneously and tracked using hand-held flexible three-element Yagi aerials and Sika receivers (Biotrack Ltd., Wareham, UK) and captured 4–6 days after deployment to download the data and recharge the GPS tag battery for further data collection whenever needed. The procedure was approved by the Institutional Animal Care and Use Committee, Qatar University (reference number: QU-IACUC 008/2012). Permission to conduct the fieldwork in Al Reem Biosphere Reserve was obtained from the General Directorate of Nature Reserves (S2 File), and no endangered species or habitats were involved in the study.

All GPS tags were programmed to record the animals’ locations once every 15 minutes during the night (18:00 h– 06:00 h) and once per hour during the day (06:00h – 18:00h) (S1 File). This schedule was designed to provide a detailed monitoring of the hedgehogs’ movement during the night (their active period) and minimize the failed attempts by the tag to acquire a location during the day when the animals are resting in shelters, which would reduce the battery life and thus fix success substantially [26]. Sampling over several nights at constant intervals also allowed us to overcome problems with autocorrelation [27, 28].

### Home range calculation and data analysis

Location data from individual hedgehogs were used to calculate home range size and daily distances travelled. Incremental analysis was performed on each home range based on 100% Minimum Convex Polygons (MCP100) and 95% Kernel Contours (K95) to determine the minimum number of locations needed for home ranges to reach an asymptote [27, 29]. Once location data passed the initial screening, home range sizes were estimated using MCP100 and K95. Because those methods may overestimate home range size when outlier locations are included [27, 30, 29], core areas (areas of intensive use) were also estimated using 50% home ranges (MCP50 and K50). The distance that an animal travelled during the night was estimated by summing the straight line distances between each location (*t*) and location (*t+1*) between 18:00 and 06:00 where (*t*) is a scheduled time for a fix and (*t+1*) is the time for the next scheduled fix (i.e. 15 min after (*t*). To investigate the spatial overlap patterns between individual hedgehogs, percentage overlap of home ranges was conducted using whole areas (MCP100 and K95) and core areas (MCP50 and K50). Overlap analysis was used to determine the percentage overlap between each pair of home ranges within (male-male and female-female) and between (male-female and female-male) sexes for the two sites separately. All location data analysis was done using Ranges8 software (v2.5, Anatrack Ltd., Warehan1, UK: Kenward et al. 2008 [31]).

General Linear Models were used to test for the effect of habitat (reserve versus farms) and sex on the hedgehog home-range size. Sex, body weight, and site were included in the model. The relationship between home range size and body weight was tested using linear regression. Differences in hedgehog body weights between the two sites and sexes were compared using a t-test. Possible differences in percent overlap in home ranges (MCP50, MCP100, K50, and K95) between neighboring individuals, sites, and sex combinations (MM, FF, MF, FM) were analyzed using ANOVA. All statistical analyses were carried out using SYSTAT 13 (Systat Software Inc., San Jose, USA).

## Results

### Population densities and body weights

Total hedgehog captures were 42 (27 ♂ and 15 ♀) in the farms area and 30 (19 ♂ and 11 ♀) within the reserve area. Hedgehog densities were estimated at 33.1 individuals/km^2^ within the farms area and 13.1 individuals/km^2^ within the reserve area. Sex ratio seemed biased (though not significantly) toward males in both sites at 1.8♂:1♀ in the farms area (Chi-squared test: χ^2^ = 2.6, p = 0.1) and 1.73♂:1♀ in the reserve area (χ^2^ = 1.6, p = 0.2). Body weights were significantly larger in the farms area (mean body weight ±SE was 417.71 ±12.77 g, n = 24) than those in the reserve area (376.37±12.71 g, n = 22) (t-test: t = 2.3, df = 44, p = 0.027). Although females were heavier than males on average in both sites, the differences were not statistically significant. In the reserve area, body weights averaged 360±20.11 g for males (n = 10) and 395±15.54 g for females (n = 12), (t-test: t = 1.35 df = 18 p = 0.196), whilst in the farms area, weights averaged 401.25±8.86 g for males (n = 12) and 434.17±23.53 g for females (n = 12), (t-test: t = 1.31 df = 14 p = 0.211).

### Incremental analysis

A total of 46 home ranges of adult hedgehogs were mapped based on 11190 usable GPS fixes with the average fix success rate of 62% (46% for the GPS BUG and 79% for the GPS PinPoints, 80% in the reserve area and 61% in the farms area). The number of fixes recorded per animal ranged between 70 and 526 tracked over 3–9 days, all of which were included into the analyses after screening by incremental analysis. The number of fixes did not have a significant effect on home range size in 100% MCP based on either pooled sample set (Linear Regression: F_1,44_ = 0.21, p = 0.65, n = 46) or within each site (reserve area: F_1,20_ = 0.181, p = 0.19, farms area: F_1,22_ = 0.08, p = 0.77). Incremental analysis suggested that an average of 115 (±7 SE) fixes (minimum of 45) were required to reach a reliable determination of home range size.

### Home range size

**Fig 3:**
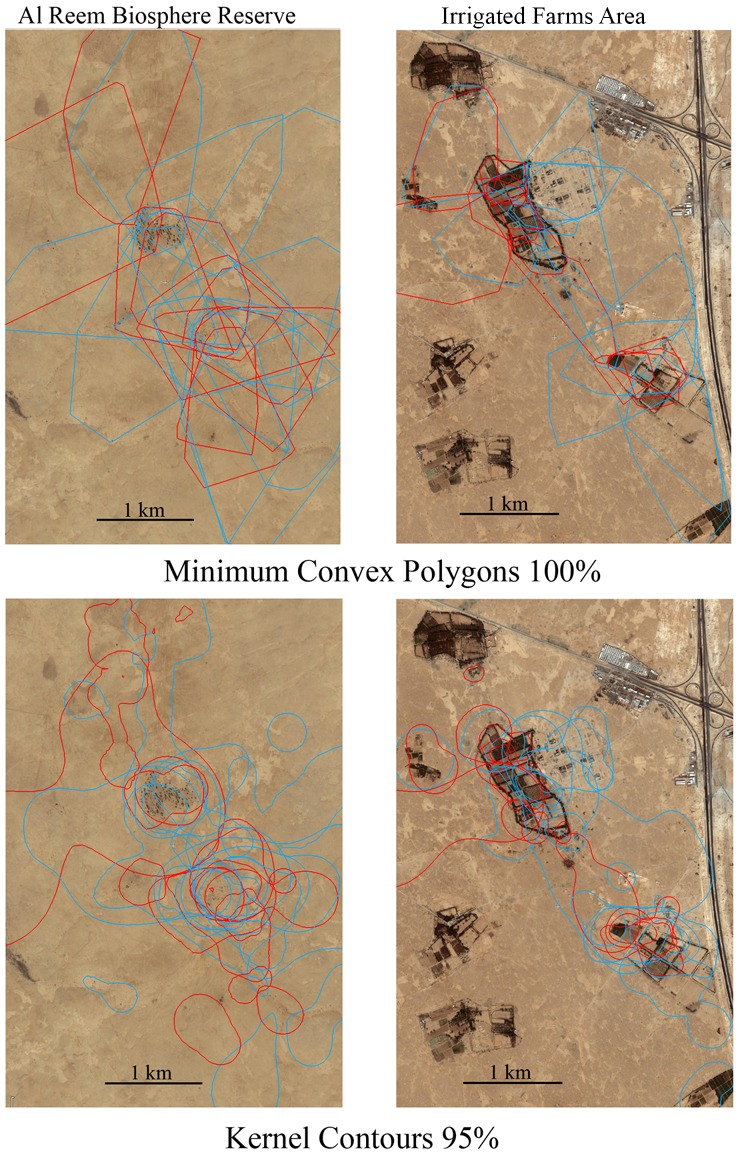
Home ranges of male (blue) and female (red) Ethiopian hedgehogs expressed as minimum convex polygons and Kernel contours from Al Reem Biosphere Reserve and the irrigated farms area.

**Fig 4:**
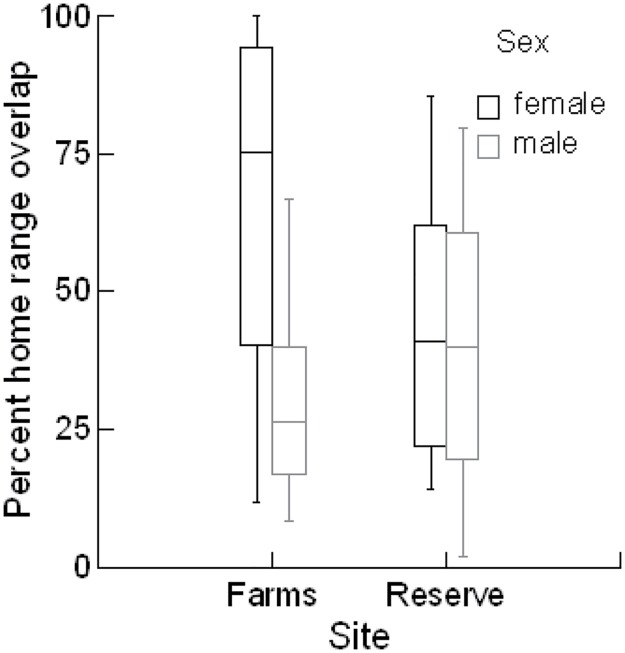
Percent overlap of male and female Ethiopian hedgehogs home ranges (expressed as minimum convex polygons) with rawdhats and farms in Al Reem Biosphere Reserve and the irrigated farms area.

Hedgehog home ranges varied in size and shape between study sites and sexes (Table 1, Fig 3). Home ranges were significantly larger in the reserve area than in the farms area, except K50 (Table 2). Home range sizes for males were significantly larger than those of females based on MCP100 and MCP50 but not K95 or K50 (Table 2). On the other hand, body weight did not have a strong influence on home range size (Table 2). Regression analysis showed that body weight did not affect home range size for either a pooled sample set (MCP100: F_1,44_ = 3.76, p = 0.059, Kernel95: F_1,44_ = 1.46, p = 0.23) or for each site separately (reserve area: MCP100 F_1,20_ = 0.003, p = 0.95, farms area: MCP100 F_1,22_ = 2.88, p = 0.1). On average, hedgehog home ranges (MCP100) spanned over 1.46 farms in the farms area (1.42 in ♀♀, 1.50 in ♂♂) and 1.90 rawdhats in the reserve area (1.70 in ♀♀, 2.08 in ♂♂) (see Fig 3), there was no statistically significant differences between the sites and sexes (ANOVA Site F_1,42_ = 2.15, p = 0.074, Sex F_1,42_ = 0.97, p = 0.33, Site × Sex F_1,42_ = 0.4, p = 0.53). Hedgehog home ranges (MCP100) overlapped with farms in the farms area by an average of 48.6% (range: 11.7–100%, average: 67.4 in ♀♀, range: 8.4–66.7%, average: 29.7 in ♂♂, t-test: t = 3.49, df = 22, p = 0.002), and with rawdhats by 42.1% in the reserve area (range: 14.3–85.4%, average: 43.8 in ♀♀, range: 1.77–79.8%, average: 40.4 in ♂♂, t-test: t = 0.30, df = 20, p = 0.76). Female home ranges overlapped in greater proportions with the farms (or rawdhats) than did those of males (ANOVA Site F_1,42_ = 0.71, p = 0.405, Sex F_1,42_ = 7.05, p = 0.011, Site × Sex F_1,42_ = 4.9, p = 0.032), and this trend is very strong in the farms area (Fig 4).

**Table 1:** Summary of Ethiopian hedgehog home range size estimates.

		MCP100	MCP50	K95	K50
**Reserve area (n = 22)**	Mean ± SE (ha)	194.6 ±23	28.5 ±5.8	128.1 ±23.7	30.3 ±6.6
	Range of values (ha)	61.2–515	0.5–91	30–472	5–108
**Farms area (n = 24)**	Mean ± SE (ha)	72.7 ±11.9	10.9 ±2.9	57.3 ±12.8	15.4±3.8
	Range of values (ha)	9.3–249	0.7–61	5.3–296.3	2–91 ha

**Table 2:** Results of General Linear Model ANOVA evaluating the influence of sex, site, and body weight on home range size and travel distance of Ethiopian hedgehogs.

Source	Type III SS	df	Mean Squares	F-Ratio	p-Value
**Minimum Convex Polygons 100% (n = 46, R = 0.68, R^2^ = 0.46)**					
**Sex**	53,264.056	1	53,264.056	8.216	0.007
**Site**	142,902.976	1	142,902.976	22.044	0.000
**Body weight**	0.796	1	0.796	0.000	0.991
**Sex × site**	1,059.133	1	1,059.133	0.163	0.688
**Error**	265,787.347	41	6,482.618		
**Kernel contours 95% (n = 46, R = 0.42, R^2^ = 0.18)**					
**Sex**	13,251.222	1	13,251.222	1.627	0.209
**Site**	48,258.096	1	48,258.096	5.925	0.019
**Body weight**	19.362	1	19.362	0.002	0.961
**Sex × site**	26.208	1	26.208	0.003	0.955
**Error**	333,962.104	41	8,145.417		
**Minimum Convex Polygons 50% (n = 46, R = 0.49, R^2^ = 0.24)**					
**Sex**	2,116.262	1	2,116.262	4.777	0.035
**Site**	3,566.590	1	3,566.590	8.051	0.007
**Body weight**	286.558	1	286.558	0.647	0.426
**Sex × Site**	4.512	1	4.512	0.010	0.920
**Error**	18,163.165	41	443.004		
**Kernel contours 50% (n = 46, R = 0.4, R^2^ = 0.16)**					
**Sex**	2,156.581	1	2,156.581	3.439	0.071
**Site**	1,999.637	1	1,999.637	3.189	0.082
**Body weight**	3.715	1	3.715	0.006	0.939
**Sex × Site**	12.528	1	12.528	0.020	0.888
**Error**	25,711.348	41	627.106		
**Mean daily distance travelled (n = 46, R = 0.75, R^2^ = 0.56)**					
**Sex**	18,200,878.045	1	18,200,878.045	13.633	0.001
**Site**	50,087,663.799	1	50,087,663.799	37.518	0.000
**Body weight**	2,596,049.343	1	2,596,049.343	1.945	0.171
**Sex × site**	947,628.856	1	947,628.856	0.710	0.404
**Error**	54,736,648.195	41	1,335,040.200		

### Distance travelled

**Fig 5:**
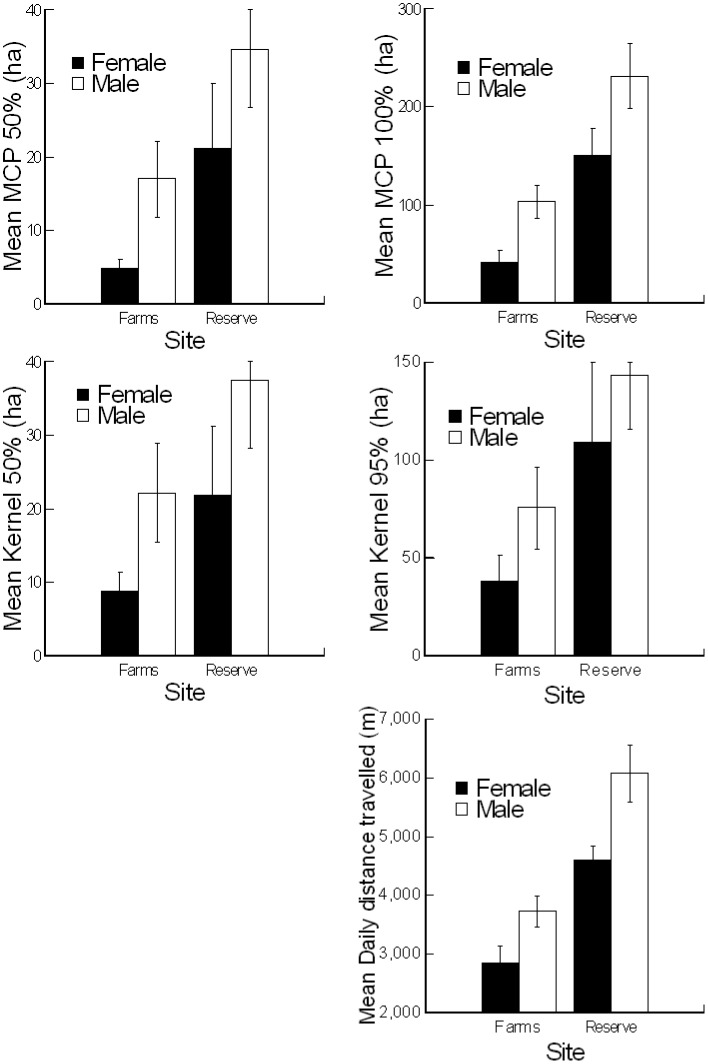
Home range sizes and daily distance travelled (mean ± SE) of male and female Ethiopian hedgehogs from two sites in northern Qatar.

Hedgehogs travelled greater distances in the reserve area (average daily distance travelled = 5402±320 m, n = 22, range: 3343–9252 m) than in the farms area (3286±220 m, n = 24, 1475–5100, Table 2, Fig 5). Average daily distance travelled was larger for males than females (Table 2, Fig 5), whilst body weight had no significant effect on daily distance travelled based on either a pooled sample (Table 2) or two sites being analyzed separately using regression analysis (reserve area: F_1,20_ = 0.71, p = 0.4, farms area: F_1,22_ = 0.3, p = 0.62).

### Home range overlap

**Fig 6:**
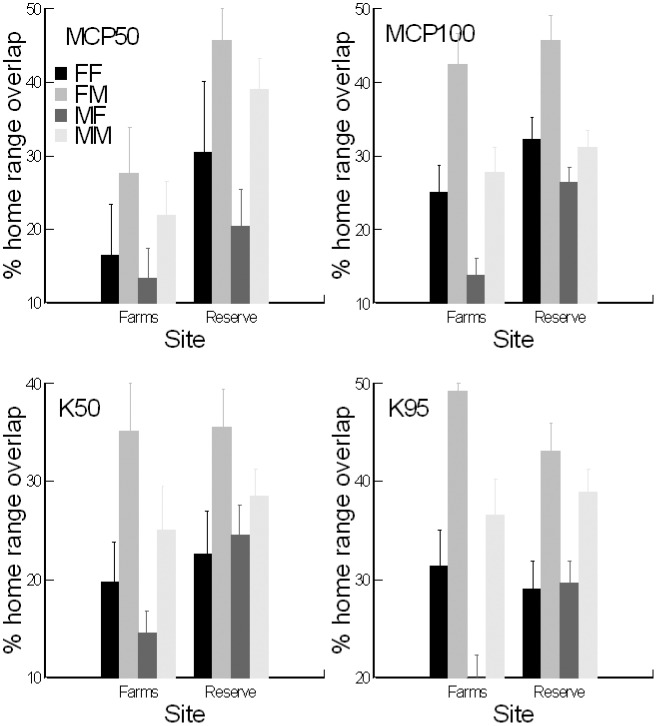
Percent home range overlap (mean ± SE) of hedgehogs in the two sites. FF female with another female, FM female with a male, MM male with another male, MF male with a female.

All animals of both sexes overlapped in their home ranges with those of other animals. However, percent overlaps decreased when core areas (MCP50, n = 208 combinations of overlap and Kernel50, n = 370) were considered in comparison to the total home range (MCP100, n = 612 and K95, n = 615) (Fig 6). Home range overlap was proportionally larger in the reserve area than in the farms area based on MCP50 and 100 but not Kernel estimates (Table 3). Percent of home range overlap was significantly different among the four sex combinations using all measures (Table 3) but not the number of overlaps among the four combinations for each home range estimate (Friedman Test: S = 3.947 df = 3 p = 0.27, S = 6 df = 3 p = 0.11, S = 5.84 df = 3 p = 0.12, S = 3.95 df = 3 p = 0.27 for the MCP50, MCP100, K50, and K95, respectively), although, we did not monitor all individuals known to be present in the areas. The combination with the lowest percent combination was always the MF (male in female), whereas the one with the highest percent overlap was consistently the FM (female in male) combination (Fig 6). This pattern was similar for all estimates.

**Table 3:** Results of General Linear Model ANOVA evaluating the influence of site, and four sex combinations on percent home range overlap in Ethiopian hedgehogs.

Source	Type III SS	df	Mean Squares		F-Ratio	P-Value
**MCP50, n = 208, R = 0.35, R^2^ = 0.12**						
**Site**	8,426.72	1	8,426.72		9.515	0.002
**Combination**	10,686.85	3	3,562.28		4.022	0.008
**Site × Combination**	935.92	3	311.97		0.352	0.788
**Error**	177,133.47	200	885.67			
**MCP100, n = 612, R = 0.34, R^2^ = 0.11**						
**Site**	6,274.608	1	6,274.608		9.394	0.002
**Combination**	43,989.354	3	14,663.118		21.952	0.000
**Site × Combination**	2,173.171	3	724.390		1.084	0.355
**Error**	403,443.230	604	667.952			
**K50, n = 370, R = 0.24, R^2^ = 0.06**						
**Site**	1,375.515	1		1,375.515	2.121	0.146
**Combination**	12,669.940	3		4,223.313	6.512	0.000
**Site × Combination**	1,094.125	3		364.708	0.562	0.640
**Error**	234,769.047	362		648.533		
**K95, n = 615, R = 0.3, R^2^ = 0.1**						
**Site**	111.958	1		111.958	0.162	0.687
**Combination**	39,120.424	3		13,040.141	18.875	0.000
**Site × Combination**	5,217.495	3		1,739.165	2.517	0.057
**Error**	419,349.934	607		690.857		

## Discussion

This study provides the first investigation of space use in the Ethiopian hedgehog, *Paraechinus aethiopicus*, based on the largest number of individual home ranges of free-ranging hedgehogs ever monitored in a single study (Table 4). Habitat richness (i.e. food availability) was shown to affect home-range size in a number of species [32, 33, 34]. Our results demonstrated that hedgehog home ranges and daily distance travelled varied significantly between areas of different land use practices and between sexes. Specifically, home ranges were larger in natural desert habitats than irrigated farms and for males than females despite no sexual size dimorphism. Individual home ranges overlapped with several others of both sexes. Body weight was not a predictor of home range size in this study within and among sexes or habitats. Our results show that hedgehogs living in more natural arid environments in the reserve area were almost 10% lighter in body weight, and exhibited larger home ranges than those living in the farms area, this is likely related to the lower productivity (i.e. less energy intake) of the former habitat, as well as hedgehogs having to travel more (i.e. more energy expenditure) (see Table 2 and Fig 5).

**Table 4:** Summary of known home range sizes for species of hedgehogs using 100% MCP estimates.

Location	Species	Reference	n	Sex	Home range (ha)
United Kingdom, golf course surrounded by private gardens	*Erinaceus europaeus*	Reeve (1982) [35]	6	♂	32±8.9
			7	♀	10±2.2
Sweden, abandoned farmland	*E*. *europaeus*	Kristiansson (1984) [36]	5	♂	46.5±15.8
			6	♀	19.7±8.4
Italy, Mediterranean maquis region	*E*. *europaeus*	Boitani and Reggiani (1984) [37]	9	♂	57.13±36.6
			5	♀	29.1±20.1
Israel, suburban area	*E*. *europaeus concolor*	Schoenfeld and Yom-Tov (1985) [38]	4	♂	1.56±0.82
			3	♀	1.58±1.25
Israel, suburban area	*Hemiechinus auritus*	Schoenfeld and Yom-Tov (1985) [38]	5	♂	4.97±3.34
			6	♀	2.85±1.4
United Kingdom	*E*. *europaeus*	Morris (1988) [39]	5		10.0–40.0
Mongolia, semiarid steppe and grasslands	*Mesechinus dauuricus*	Murdoch et al. (2006) [40]	5	♂	462.46 ± 125.62
			2	♀	323.38 ± 119.21
Denmark, arable land, forests, and grassland	*E*. *europaeus*	Riber (2006) [41]	4	♂	96 ± 24
			4	♀	26 ± 15
United Kingdom, residential area	*E*. *europaeus*	Dowding et al. (2010) [42]	19	♂	2.87 ± 1.74
			19	♀	0.77 ± 0.40
Ireland, rural area	*E*. *europaeus*	Haigh et al. (2011) [43]	4	♂	56.0±0.67
			3	♀	16.5±0.49
Mongolia, semiarid steppe and grasslands	*M*. *dauuricus*	Zapletal et al. (2012) [44]	8		113.15–2,171.97
Finland, urban area	*E*. *europaeus*	Rautio et al. (2013) [45]	4	♂	97.9±6.1
			3	♀	55.2±17.1
Spain, agricultural plots and pine stands	*Atelerix algirus*	García-Rodríguez and Puig-Montserrat (2014) [46]	7	♂	22±13
			7	♀	17.1±3.7
**Qatar**: irrigated farms desert reserve	*Paraechinus aethiopicus*	**Present study**	12	♂	103.4±17.1
			12	♀	42±11.5
			12	♂	231.2±33.15
			10	♀	150.6±26.86

### Spacing patterns of Ethiopian hedgehogs

Mammals living in habitats with low primary productivity are expected to have larger home ranges to meet their bioenergetic demands [47]. Ethiopian hedgehogs in Qatar exhibited the second largest home range size of any hedgehogs studied so far, exceeded only by the Daurian hedgehog, *Mesechinus dauuricus* from Mongolia (Table 4). Based on our GPS tracking results, the hedgehogs aggregated in areas that appeared to have high vegetation cover represented by the irrigated farms in the farms area and the ‘rawdhats’ in the reserve area (Fig 3). They lived in the farms area at more than twice the density and with less than half the home range area compared to those in the reserve area, probably a result of differences in resource availability. The hedgehog populations were structured in the form of small, open metapopulations within areas of higher productivity (farms and ‘rawdhats’) separated by open bare spaces. The individuals (mostly males) moved in and out of those populations in search of resources.

Although there was no significant sexual size dimorphism in terms of body weight, males’ home ranges were larger than those of females. If body size—a metabolically based parameter—was the best predictor of home range size, predicted male home range size is given by the following equation [48, 49].

$$\text{male home range size}=\frac{\text{female home range size }\times \;{\left(\text{male weight}\right)}^{0.75}}{{\left(\text{female weight}\right)}^{0.75}}$$

This assumes that resources used by females and males have the same pattern of availability and that the sexes are not cohabiting or, if they are, that they are not depleting each other’s resources [50]. As little information is available concerning these two assumptions for hedgehogs, we assume that they are plausible. Then, by following the foregoing equation, the predicted size of a male's home range would be c. 94% and c. 93% of that of females for farms area and reserve area, respectively. However, observed average male home range size was c. 1.5 times as large as that of females in the reserve area, and c. 2.5 times in the farms area. The spatial behavior of males is driven by the need to increase their fitness, which required moving greater distances to find more mates (e.g. [51]). As a solitary small mammal, receptive females are expected to be widely dispersed over the low productive, patchy habitats of the desert [14, 16, 52]. This pattern is expected in a non-territorial mammal with a promiscuous mating system [53, 54, 55, 56]. Ethiopian hedgehogs have a 6-month breeding season between February and July with two peaks of mating and 2–3 litters [14, 57, 58]. Our findings suggest that during the study periods, which were in the breeding season, male *P*. *aethiopicus* roamed over large areas to court as many females as possible to increase their reproductive success. This sexual dimorphism in home range size is consistent with previous observations in other small insectivorous mammals, including hedgehogs (e.g. Algerian hedgehog, *Atelerix algirus* [46]; greater hedgehog tenrec, *Setifer setosus* [56]; Daurian hedgehogs, *Mesechinus dauuricus* [44]; European hedgehog, *Erinaceus europaeus*, [35, 37, 41, 43, 45, 59, 60]; short-beaked echidnas, *Tachyglossus aculeatus*, [61], see Table 3).

Our results also suggest that the activity centers (or core area) of hedgehog home ranges of both sexes were restricted to the patches (or ‘islands’) of higher-quality habitats (i.e. the rawdhats and farms) that provide both foraging and nesting opportunities (see Kernel home ranges in Fig 3). However, both males and females do travel outside of those ‘islands’ (see MCP home ranges in Fig 3). This roaming behavior appears to be more detectable in males as their ranges spanned over more farms/rawdhats and were significantly larger than those of females based on MCP methods (e.g. roaming area) whilst they were not so based on Kernel methods (e.g. core area), (Table 3). The results showed that male home ranges spanned over more farms and/or rawdhats, but the percentage of overlap between home ranges and farms and/or rawdhats was greater for females. In other words, it is likely that the size of the core area for males for survival is similar to that for females, but males tend to roam out further from this core area presumably seeking mating opportunities with as many receptive females as possible. Due to the males’ larger home ranges, females’ ranges had a greater area of overlap with those of males than with other females (Table 3, Fig 6). This may increase the males’ chances to encounter receptive females. Individual hedgehog home ranges overlapped with those of other animals of both sexes, even in core areas, this is consistent with previous suggestions that hedgehogs are not territorial (e.g. [16]). The apparent lack of territoriality may reduce the cost associated with territorial defense and allow the males to roam further beyond their core areas in search of receptive females [51, 62].

### Land use change and its effects on hedgehogs

The results suggest that the sites influenced hedgehog home range size more than any other variable that we analyzed, including sex (Tables 1 and 2). The presence of irrigated farms in an arid environment clearly increased the food and nesting resources, and influence the spatial patterns of Ethiopian hedgehogs. In such areas, both males and females appeared to become heavier and maintained smaller home ranges in comparison to those living in more natural arid environments. A female’s heavier weight and small home range size probably resulted from the increased availability of water, food and shelter, as well as their distribution patterns. Modern agricultural activities in the region have increased the availability of some key resources, including water, food (e.g. invertebrates, bird eggs, frogs and small geckos), and shelter for local wildlife, as well as increasing the habitat heterogeneity which provides plenty of shelter and refuge areas [17, 63, 64]. Such environmental enrichment often provides congregation or ‘rendezvous’ spots such as garbage dumbs and prey ‘hot spots’ that animals associate with the presence of food or individuals may have better chances of finding mates, communicating and/or exchanging information, and influenced space use of hedgehogs [32, 64]. Our results showed that female home range size was significantly smaller but bodyweight greater in the farms area than that in the more natural desert habitat in the reserve area. Considering that female home range size is largely determined by the availability of food and shelter (e.g. [51]), this may suggest that such resources are available at a greater density in the farms area. Also, female movement in the farms area appears to be dependent on the distribution of irrigated farms and mostly restricted within their boundaries or between closest farms (Fig 3), further supporting the idea that irrigated farms provide resource centers for female hedgehogs. Then, a male’s optimal weight, home range size, and movement may be determined by the resources and its reproductive success, which is mainly influenced by spatial patterns and mating opportunities with the females. Perhaps, as expected, male hedgehog movements were also dependent on the presence of the farms (Figs 3 and 4) probably not only because of the increased availability of food and shelter in farms, but also because of the higher concentration of females there [51, 53, 65, 66]. However, our results suggest that rawdhats probably had always played the similar role as resource centers for local hedgehogs (and other organisms) in arid environments before the more productive modern irrigated farms replaced them. Our results also suggested that free-ranging male Ethiopian hedgehog in the open desert can keep a home range more than twice as large as that in the farms area. Such a large home range may benefit the male’s reproductive success, and yet they did not do so in the farms area (Fig 5). This may be due to the larger population size in the farms area, it is also possible that maintaining a large home range in the farms area is costly for the males in terms of survival, further research may provide answers.

### Conservation implications

Degradation and alteration of the natural environments through land use activities (i.e. agricultural expansion) have major consequences on wildlife and movement patterns [67], particularly in arid environments where the transformation of the land from a dry land to ‘islands of fertility’ is extreme. As spatial expression of animal behavior, home range and daily movement correspond with resource availability and distribution, including potential mates, and directly influence their survival and reproduction [47, 68, 69, 70]. As far as Ethiopian hedgehogs in Qatar are concerned, our results may suggest that the recent change in agricultural land use is not immediately threatening its survival although it undoubtedly influences its behavior and ecology. However, this change in agricultural land use may have resulted in a shift of optimal body weight and home range size of this desert-adapted small hedgehog towards heavier weight and smaller home ranges. Considering the growing human population and facing the possibility of food shortages in the Middle Eastern and North African region, it would be extremely difficult, politically and socially, to keep arid environments pristine. Consequently, the region’s biodiversity will be influenced by changing anthropogenic land use. This increases the need to understand the responses of indigenous wildlife to such changes, and to try to minimize anthropogenic effects on these species.

Land use and agro-environmental policies should be implemented to balance farming intensity and habitat conservation for wildlife. In general, biodiversity-friendly farming schemes should increase habitat heterogeneity and/or the presence of islands or corridor reserves to support the local wildlife [63, 64, 71, 72]. We recommend that such biodiversity-friendly agro-environmental schemes be introduced in the Middle East to minimize the impacts of agriculture on wildlife species, space use, and habitat utilization patterns, which are key factors in shaping aspects of the reproductive biology and mating systems [22, 73]. The limited resource availability in the Ethiopian hedgehog’s arid environment resulted in larger home ranges and distances travelled that centered on irrigated farms and rawdhats, it also drove the males to invest their much-needed energy into the search of receptive females across larger areas to increase their mating opportunities. The space use promiscuous mating system in Ethiopian hedgehogs reveals an interplay of space use, habitat utilization, and reproductive strategy. The results add to our knowledge of mammalian space use and reproductive investment, particularly to the behavioral ecology of the little-known Ethiopian hedgehogs. Further research is necessary to increase our understanding of this desert-adapted small hedgehog, including the effect of space use on its reproductive strategies such as mate choice [74].

## Supporting information

S1 File Minimal data set showing the schedule of data collection. (CSV)

S2 File Permission letter from the General Directorate of Nature Reserve to conduct the study in Al Reem Biosphere Reserve. (PDF)
