# Supplementary material for: Hedgehogs on the move: Testing the effects of land use change on home range size and movement patterns of free-ranging Ethiopian hedgehogs
Source: PLoS One. 2017 Jul 26;12(7):e0180826. doi: 10.1371/journal.pone.0180826 (PMC5528257; doi:10.1371/journal.pone.0180826)
Supplement: S2 File — (PDF) [file pone.0180826.s002.pdf]

قطــاع المحميــات الطبيعيــة

GENERAL DIRECTORATE OF NATURE RESERVES

نحميهـا ... لننعـم ... ونغنـم

دولة قطر
قطاع المحميات الطبيعية

الإشارة : ٣٤١/١٥/٠٠١

التاريخ : ٢٠١٥ / ٣ / ١

الدكتورة/ فاطمة عمار النعيمي المحترم،،

رئيس قسم العلوم البيولوجية والبيئية

جامعة قطر

الدوحة

السلام عليكم ورحمة الله وبركاته،،،

الموضوع : إجراء بحث بيئي في محمية الريم –جامعة قطر

يهديكم قطاع المحميات الطبيعية أطيب تحيّة،بالاشارة الى كتابكم (مرفق) بخصوص الموضوع اعلاه وفي اطار التعاون المشترك نحيطكم علما بأنه لامانع لدينا من اجراء البحث في محمية الريم .

عليه يرجي التكرم بالإيعاز لجهة الاختصاص لديكم للتنسيق مع السيد/ ناصر الكبيسي ، هاتف رقم (٥٥٨٥٥٥١٠).

وتفضلوا سيادتكم بقبول فائق الاحترام والتقدير،،

نــواف جبــر النعيمي

مدير مكتب رئيس القطاع

المرفقات :
- نسخة من الكتاب المشار اليه اعلاه .

نسخة إلى :
- السيد/مدير ادارة الحمى .

من محمياتنا ...

Fax : +974 4420 77 34 : فاكس

P.O.Box : 18859 - Doha - Qatar : ص . ب

E-mail : gdnr@nrqa.org : بريد الكتروني

محمية الريم AL Reem Nature Reserve

محمية الذخيرة AL Thajerah Nature Reserve

محمية خور العديد Khor Al Uddid Nature Reserve

كلية الآداب والعلوم
College of Arts and Sciences
QATAR UNIVERSITY جامعة قطر

قسم العلوم البيولوجية والبيئية
Department of Biological & Environmental Sciences

قطاع المحميات الطب
الشؤون الإدارية والما
رقم الوارد : ١٠٢/١٥/٢٠١٥
التاريخ : ٢٥/٢/٢٠١٥
توقيع المستلم :

السيد رئيس قطاع المحميات الطبيعية
المكتب الهندسي الخاص
صندوق بريد 18859
فاكس 44207734
الدوحة - قطر

الموضوع: إجراء بحث بيئي في محمية الريم - جامعة قطر

السلام عليكم و رحمة الله و بركاته

نرجو التكرم بتمديد الموافقة بالسماح للدكتور نوبي ياماغوشي -و الذي يعمل كعضو هيئة تدريس في قسم العلوم البيولوجية و البيئية في جامعة قطر- و فريقه بالدخول و العمل في محمية الريم لإجراء بحوث ميدانية لدراسة سلوك و بيئية القنفذ الأثيوبي حتى نهاية العام 2015. علما بأنهم قد حصلوا على الموافقة للعمل خلال العام 2014 بالإشارة لكتابكم بتاريخ 2014/4/7 (مرفق).

كما يرجى العلم بأن البحث المدعوم من الصندوق القطري لرعاية البحث العلمي (بحث رقم 5-083-1-019) يستغرق ثلاث سنوات (سبتمبر 2013 – أغسطس 2016) سيتضمن إصطياد القنافذ البرية الساكنة للمنطقة خلال ساعات الليل ومن ثم إطلاقها بعد تركيب أجهزة إستشعار عن بعد عليها و ذلك لدراسة أعدادها في المنطقة و تحركاتها الطبيعية و تزاوجها. كما أن فريق البحث و المكون من الدكتور نوبي ياماغوشي و الدكتور محمد عدنان أبوبكر و الأستاذ إفان منديز يمتلكون الخبرة اللازمة للتعامل مع هذه الكائنات، كما أنهم على علم بطبيعة المنطقة. في حال الحاجة للاستفسار عن تفاصيل البحث، يرجى الاتصال مع منسق المشروع: د. محمد أبوبكر على هاتف 55124888، بريد الكتروني mabubaker@qu.edu.qa.

و تفضلوا بقبول فائق الاحترام و التقدير،،

رئيس قسم العلوم البيولوجية و البيئية

د. فاطمة عمار النعيمي

السيد / الشيخ عبدالله الثاني
أرجو العمل اللازم
للتجديد التصريح
٢٥/٢/٢٠١٥

صندوق بريد: ٢٧١٣ الدوحة - قطر هاتف: ٤٤٠٣٤٥٧٠ - ٤٤٠٣٤٥٣٤ (+٩٧٤) فاكس: ٤٤٠٣٤٥٣١ (+٩٧٤)
P.O.Box: 2713 Doha - Qatar Tel.: (+974) 4403 4570 - 4403 4534 Fax: (+974) 4403 4531 email: biology@qu.edu.qa www.qu.edu.qa/artssciences

الإشارة : ٢٨٤/١٤١/٤
التاريخ : ٧/ ٤/ ١٤٠٢م

السيد/ رئيس قسم العلوم البيولوجية والبيئة المحترم،،
كلية الآداب والعلوم
جامعة قطر
الدوحة

السلام عليكم ورحمة الله وبركاته،،،

الموضوع : إجراء بحث بيئي في محمية الريم – جامعة قطر

يهديكم قطاع المحميات الطبيعية أطيب تحية، وبالإشارة إلى كتابكم رقم:١٩٤٥(مرفق) بخصوص الموضوع أعلاه وفي اطار التعاون المشترك نحيطكم علماً بأنه لا مانع لدينا من اجراء البحث في محمية الريم.

عليـه يرجى من التكرم بالإيعاز لجهة الاختصاص لديكم للتنسيق مع السيد/ ناصر الكبيسي، جوال رقم: ٥٥٨٥٥٥١٠.

وتفضلوا سيادتكم بقبول فائق الاحترام والتقدير،،،

نواف جبر النعيمي
مدير مكتب رئيس القطاع

نسخة :
- السيد/ مدير إدارة الحمى.

فاكس : +974 4420 77 34
ص . ب : 18859 - Doha - Qatar Box :
بريد إلكتروني : gdnr@nrqa.org ail :

كلية الآداب والعلوم
College of Arts and Sciences
QATAR UNIVERSITY

قسم العلوم البيولوجية والبيئية
Department of Biological & Environmental Sciences

قسم العلوم البيولوجية والبيئية
Dept. of Biological & Environmental Sciences
17 FEB 2014
رقم الصادر: 1945

قطاع المحميات الطبيعية
الشؤون الإدارية والمالية
رقم الوارد: ٧١/١٤/ح
التاريخ: ٢٠١٤/٢/٢٣
توقيع المستلم:

السيد رئيس قطاع المحميات الطبيعية
المكتب الهندسي الخاص
الدوحة

الموضوع: إجراء بحث بيئي في محمية الريم – جامعة قطر

السلام عليكم ورحمة الله وبركاته ...

نود أن نحيطكم علماً بأن الدكتور نوبي ياماغوشي و الذي يعمل كعضو هيئة تدريس في قسم العلوم البيولوجية و البيئية في جامعة قطر قد حصل على دعم من الصندوق القطري لرعاية البحث العلمي (بحث رقم 019-1-083-5) لإجراء بحوث ميدانية لدراسة سلوك و بيئة القنفذ الأثيوبي. نرجو السماح له بالعمل في منطقة روضة الفرس و تسهيل مهمته ما أمكن، كما نرجو السماح له بالدخول و العمل في محمية الريم و ذلك لمقارنة النتائج من روضة الفرس (و التي تتميز بانتشار المزارع الخاصة) مع بيئة طبيعية وذلك خلال الفترة Feb. و Dec. 2014.

كما يرجى العلم بأن البحث سيتضمن اصطياد القنافذ البرية الساكنة للمنطقة خلال ساعات الليل ومن ثم إطلاقها بعد تركيب أجهزة إستشعار عن بعد عليها وذلك لدراسة أعدادها في المنطقة و تحركاتها الطبيعية وتزاوجها. كما أن فريق البحث والمكون من الدكتور نوبي ياماغوشي و الدكتور محمد عدنان أبوبكر والأستاذ إفان منديز يمتلكون الخبرة اللازمة للتعامل مع هذه الكائنات، كما أنهم على علم بطبيعة المنطقة و قد قاموا بإجراء الإتصالات اللازمة مع المزارع الخاصة للسماح لهم بالعمل. للاستفسار عن تفاصيل البحث، يرجى الاتصال مع منسق المشروع: د. محمد أبوبكر على هاتف 55124888، بريد الكتروني mabubaker@qu.edu.qa.

و تفضلوا بقبول فائق الاحترام و التقدير...

أ. د. سمير الجوة
رئيس قسم العلوم البيولوجية و البيئية
